# *Bifidobacterium longum* increases serum vitamin D metabolite levels and modulates intestinal flora to alleviate osteoporosis in mice

**DOI:** 10.1128/msphere.01039-24

**Published:** 2025-02-21

**Authors:** Hongchao Wang, Gao Tian, Zhangming Pei, Xihua Yu, Yi Wang, Fuchun Xu, Jianxin Zhao, Shourong Lu, Wenwei Lu

**Affiliations:** 1 State Key Laboratory of Food Science and Resources, Jiangnan University, Wuxi, Jiangsu, China; 2 School of Food Science and Technology, Jiangnan University, Wuxi, Jiangsu, China; 3 Sinopharm Xingsha Pharmaceutical (Xiamen) Co., Ltd., Wuxi, China; 4 The Affiliated Wuxi People’s Hospital of Nanjing Medical University, Wuxi People’s Hospital, Wuxi Medical Center, Nanjing Medical University, Wuxi, Jiangsu, China; 5 National Engineering Research Center for Functional Food, Jiangnan University, Wuxi, Jiangsu, China; Kansas State University, Manhattan, Kansas, USA

**Keywords:** osteoporosis, gut microbiota, vitamin D, active vitamins

## Abstract

The elderly population is prone to osteoporosis, owing to the deterioration of the skin, liver, and kidney functions. Vitamin D (VD) supplementation has a limited effect, and VD deficiency is mostly treated with medication. Several studies have shown that the gut microbiota alters intestinal VD metabolism and that probiotic supplements can influence circulating VD levels. Therefore, in the present study, we screened a strain of *Bifidobacterium longum* FSHHK13M1 that can increase the level of VD metabolites in the fermented supernatant species *in vitro* by modeling fecal bacterial fermentation. The results showed that FSHHK13M1 intervention significantly increased the serum levels of 1,25-dihydroxy VD and osteocalcin. It activated the expression of the VDR, OPG, Wnt10b/β-catenin, and Runx2/Osterix pathways and inhibited the expression of RANKL/RANK pathway. Furthermore, there was an enhancement in the quantity of bone trabeculae and the proportion of bone volume. Concurrently, the gut microbiota in mice with osteoporosis exhibited signs of imbalance. FSHHK13M1 intervention increased the relative abundance of specific bacteria, such as *Faecalibaculum rodentium*, *Limosilactobacillus fermentum*, *Bifidobacterium pseudolongum*, and *Akkermansia muciniphila*. These results suggest that *B. longum* FSHHK13M1 alleviates retinoic acid-induced osteoporosis symptoms by modulating related genes, regulating the intestinal flora and increasing the level of active VD.

**IMPORTANCE:**

Osteoporosis is a systemic metabolic disease in which the patient’s bone mass decreases for a variety of reasons, and the microstructure of the bone tissue is altered, leading to an increase in bone brittleness and susceptibility to fracture. Osteoporosis is almost always present in the elderly population, and fractures from falls are an important predisposing factor for mortality risk in the elderly population. Supplementation is quite limited for them as they are not able to utilize vitamin D well due to declining liver, kidney, and skin functions. In the present study, a strain of *Bifidobacterium longum* probiotic was found to increase the levels of the active form of vitamin D and ameliorate osteoporosis. This may play an important role in preventing osteoporosis and reducing fracture risk in the elderly.

## INTRODUCTION

Osteoporosis is a systemic metabolic disease in which bone mass is lost. It reduced for various reasons and the microstructure of bone tissue is altered, leading to an increase in bone fragility, which can easily cause fractures in patients (1). Osteoporosis is categorized as primary or secondary. Primary osteoporosis is further categorized into senile and post-menopausal types. Common symptoms of osteoporosis include pain, shortened body length, hunchback, fractures, and decreased respiratory function. Calcium supplementation is the basic measure for the treatment of osteoporosis; however, calcium supplementation alone is not sufficient for the treatment of osteoporosis, and medication should be added according to the condition of the patient.

Currently, bisphosphonates, selective estrogen receptor modulators, estrogen, and calcitonin are primarily used for the clinical treatment of osteoporosis. These drugs effectively promoted osteoblast formation and inhibited osteoclast formation. However, all bisphosphonates affect renal function and may cause or exacerbate hypocalcemia (1). In addition, bisphosphonates can cause dysphagia, esophageal inflammation, and stomach pain. Rare atypical femoral fractures (AFFs) and osteonecrosis of the jaw (ONJ) occur in a small number of patients because of bisphosphonate use (2). There is also a documented incidence of ocular inflammation (anterior uveitis and episcleritis) (3). The selective estrogen receptor modulator, such as raloxifene, increases risk of venous thromboembolism and stroke. Estrogen increases the risk of endometrial hyperplasia and cancer in patients and increases the incidence of gallstone disease and venous thromboembolism (4, 5). The risk of the receptor activator of NF-κB ligand (RANKL) inhibitor, disumab, was similar to that of bisphosphonates. Calcitonin may cause allergic reactions, and anaphylaxis may occur in severe cases. High doses of calcitonin can lead to hypocalcemia, and there is a link between calcitonin levels and cancer risk (6). All anti-fracture therapies treat but do not cure the disease. Bone deterioration recurs sooner or later after discontinuation of medication. As many as 80%–95% of patients are discharged from the hospital after hip fracture repair without anti-fracture therapy or management program (7–9). Therefore, there is an urgent need to find a drug or treatment that is effective in relieving osteoporosis and does not lead to long-term complications or side effects in patients.

Vitamin D (VD) is essential for maintaining the physiological function of bones and for their growth. Its role in maintaining bone health and regulating mineral ion homeostasis is well documented. In addition, epidemiological evidence suggests that low VD levels are consistently associated with several common diseases, such as metabolic, cardiovascular, autoimmune, musculoskeletal, malignant, and infectious diseases (10–12). 1,25-Dihydroxy VD [1,25(OH)2D], which is the active form of VD, acts as a direct agonist that binds to VDR. VDR is important for regulating calcium homeostasis and the immune system (13). Children with VD and older adults receiving appropriate VD supplementation also play important roles in maintaining bone health and regulating mineral ion homeostasis. Appropriate supplementation of VD in children and the elderly produces direct benefits to the skeleton, such as improving muscle strength and regulating the ability of the body to regulate balance.

Patients with osteoporosis may have reduced ability to metabolize VD for several reasons. Patients with osteoporosis typically have reduced ability to metabolize VD. VD is a fat-soluble vitamin that is synthesized by skin exposure to ultraviolet light and dietary intake. VD undergoes two key metabolic steps in the body to be effective. First, VD is converted to 25-hydroxy VD [25(OH)D] in the liver (14), and then further converted into 1,25(OH)_2_D in the kidneys (15). VD metabolites enter the intestine with bile acids and are reabsorbed at the end of the ileum. Thus, diseases of the terminal ileum, general malabsorption, and other causes of shorter intestines can lead to lower serum 25(OH)D levels. Aging and decreased kidney function are the most common causes. With age, the ability of the skin to synthesize VD diminishes, resulting in decreased levels of VD stored in 25(OH)D. Simultaneously, renal decline may hinder the production of 1,25(OH)2D, further reducing VD (16). Therefore, the elderly population and patients with osteoporosis need to compensate for their reduced ability to synthesize VD through additional VD intake. A recent recommendation by the American Endocrine Society is that empirical VD supplementation and routine screening of 25(OH)D levels are not required in the general population (17). The ability to metabolize VD is more critical than increasing VD intake. In this context, increasing the level of 1,25(OH)2D has become even more critical.

In recent research, some probiotics, especially *Bifidobacterium* and *Lactobacillus*, have the ability to improve bone health (18). We conjecture that this may be related to the promotion of VD metabolism by probiotics. Therefore, we selected some species of *Bifidobacterium* genus and *Lactobacillus* genus for *in vitro* fermentation experiments, including *Bifidobacterium longum*, *Bifidobacterium adolescentis*, and *Bifidobacterium longum* subsp. *infantis*, et al. (19, 20).

In the present study, we screened a strain of *B. longum*, FSHHK13M1, that increased the levels of VD metabolites in the fermentation supernatants using an *in vitro* fecal fermentation model. We further investigated the effect of FSHHK13M1 on retinoic acid-induced (RA-induced) osteoporosis in mice using micro-computed tomography (micro-CT) and analyzed the possible mechanisms of this association by gut microbiota analysis. The results of this study may provide insights into the use of probiotics to increase VD activity against osteoporosis. It has great potential for application in the preparation of products that enhance the physiologically active function of VD and prevent or treat osteoporosis and bone loss.

## MATERIALS AND METHODS

### *In vitro* fermentation model screening strains

A quantity of 10 g of fecal material was combined with 90 mL of sterile PBS at a concentration of 1:10 (wt/vol), ensuring a complete mix and homogenization. The resulting mixture underwent filtration through a double layer of gauze to eliminate any undissolved solid particles, all within the confines of an anaerobic environment. In a 24-well plate, a fecal suspension was created, with each well receiving 1.6 mL of mGAM growth medium. Subsequently, a mixture of 0.3 mL of the fecal suspension and 0.1 mL of different bacterial was introduced into each well, followed by a 24 h incubation period under anaerobic conditions. The preparation was replicated three times for each sample. In addition, some wells received 0.4 mL of the fecal suspension for control purposes. The fermentation was carried out over a full day within an anaerobic chamber. Post-fermentation, the plate was briefly chilled on ice for 15 min to halt the process. The samples were then moved to 2 mL sterile centrifuge tubes for separation of the supernatant and pellet via centrifugation at 7,500 rpm for 15 min. The supernatant was subsequently stored at −20°C, ready for subsequent analysis of 25(OH)D and 1,25(OH)2D concentrations.

### Preparation of gavage strains for animal experiments

The *B. longum* culture was preserved in a Man–Rogosa–Sharpe (MRS) broth enriched with a 30% (vol/vol) glycerol solution, kept at a temperature of −80°C. Prior to the experimental procedure, the culture underwent a series of three successive revivals. Following centrifugation of the bacterial mixture at 8,000 × *g* for a duration of 10 min to separate the liquid phase, the remaining pellet was repeatedly washed three times using a sterile saline solution. For experimental application, the bacterial solution was further diluted to achieve a concentration of 10^9^ colony-forming units (CFU)/mL using sterile saline.

### Mice experimental design

This research utilized 32 male C57BL/6J mice, categorized as specific pathogen-free (SPF), sourced from Zhejiang Vital River Laboratory Animal Technology Co., Ltd., at 7 weeks of age and weighing approximately 20 ± 5 g. The animals were maintained in an environment with regulated temperature, ranging from 23°C ± 2°C, and humidity levels of 50% ± 10%. They had unrestricted access to a diet that conformed to national guidelines for laboratory rodents. The experimental procedures were carried out at Jiangnan University’s Animal Experiment Center in Wuxi, China. The ethical considerations of the study were reviewed and approved by the Jiangnan University Experimental Animal Ethics Committee, with the approval number JN.No20231030c1360128[515].

Mice were acclimatized for 1 week at 21°C–26°C. Then, these mice were randomly divided into four groups (*n* = 8 each): control, VD, model, and FSHHK13M1. Except for the control group, mice were orally gavaged with 200 µL of 90 mg/kg body weight/day (mg/kg bw/day) retinoic acid (RA) daily for 3 weeks to induce osteoporosis. After modeling, 24 mice were randomly allocated to three groups based on body weight: model, VD, and FSHHK13M1. The VD group received oral gavage of 0.06 µg/kg bw/day VD, and the FSHHK13M1 group received oral gavage of *B. longum* FSHHK13M1 suspension at a concentration of 1 × 10^9^ CFU/mL. The control and model groups received oral gavage of saline (200 µL/day) daily for 3 weeks. During the experiment, all the mice had free access to water and standard rodent feed.

Before the end of the study, fresh excretions from mice were collected into 2 mL sterile Eppendorf tubes and stored at −80°C for subsequent gut microbiome analysis. After anesthesia with isoflurane, mice were humanely euthanized by eye removal induced bleed-out. Blood was collected and centrifuged at 4,500×*g* for 15 min at 4°C. The serum is carefully separated and aliquoted into several sterile 100 µL microtubes and stored at −80°C. The serum is then carefully extracted from the femur of the mouse. The mouse femur and tibia are then removed and placed into 5 mL sterile, non-transparent Eppendorf tubes pre-filled with 4% paraformaldehyde (PFA) in preparation for micro-CT measurements.

### Determination of structural indices of mouse femur

Following the excision of the right tibia from each rodent and subsequent fixation in a 4% PFA, the samples were left to dry for subsequent imaging. Utilizing a micro-CT scanner model Lateta LCT200 from Hitachi-Aloka in Tokyo, Japan, the samples underwent scanning with the following settings: 90 kV voltage, 80 μA current, a radiation dose of 1,608 mGy, a field of view spanning 18 mm, and a voxel resolution of 36 µm. Each femur was meticulously rotated a full circle to ensure comprehensive data capture, which was subsequently transferred to Analyze software version 12.0 by AnalyzeDirect for three-dimensional (3D) modeling and evaluation. The analysis focused on a region of interest (ROI) extending 1 mm in thickness, commencing 0.2 mm beneath the growth plate, to ascertain various bone metrics. The pivotal parameters measured encompassed the average bone density (BM), average cortical density (CM), average trabecular density (TM), spacing between trabeculae (Tb.Sp), count of trabeculae (Tb.N), thickness of trabeculae (Tb.Th), surface area of the bone (BS), total bone volume (BV), ratio of bone volume to total volume (BV/TV), and density of bone trabecular connectivity (Conn.D).

### Biochemical analysis

Serum 25(OH)D and 1,25(OH)_2_D levels were measured using commercial mouse enzyme-linked immunosorbent assay (ELISA) kits (Beyotime Biotechnology, Shanghai, China). Serum calcium and alkaline phosphatase (ALP) levels were measured using commercial detection kits (Nanjing Jiancheng Bioengineering Institute). Osteocalcin (OCN) and procollagen type I N-propeptide (PINP) levels were measured using commercial mouse ELISA kits (E-EL-M0864, E-EL-M0233, Elabscience Biotechnology).

### RT-PCR of mouse bone metabolism related genes

The mouse femur samples were homogenized in an ice bath. RNA was extracted from the homogenate sequentially with chloroform and isopropanol, then the mixture were centrifuged at 12,000 rpm for 15 min at 4°C. The precipitates were repeatedly washed with precooled 75% ethanol and then dissolved to obtain total RNA solutions. The RNA concentration and quality were evaluated with spectrophotometry and agarose-gel electrophoresis. The total RNA from each sample was reverse transcribed to cDNA with reverse transcription kit (R433-01, Vazyme, Nanjing, China), according to the manufacturer’s instructions.

Real-time quantitative PCR was performed with SYBR qPCR Mix (Q712-02, Vazyme, Nanjing, China) in a QuantStudio 6 Flex Real-time PCR System (Applied Biosystems, Foster City, CA, USA). The PCR cycling program was as follows: pre-denaturation at 95°C for 30 s, followed by 40 cycles of denaturation at 95°C for 10 s, and annealing at 60°C for 30 s. The melting curve was increased from 65°C to 95°C by 5°C for 5 s each time. After normalization using GAPDH as an internal reference, the relative expression level of the target gene was expressed as the ratio of the expression level of the target gene to that of the corresponding gene in the model group. Primers for vitamin D receptor (VDR), Wnt10b, β-catenin, Osterix, Runx2, RANKL, and OPG are shown in Table 1. Primers were designed and synthesized by Sangon Biotech (Shanghai) Co., Ltd.

**TABLE 1:** Sequences of primers in quantitative real-time PCR

Gene name	Bidirectional primer	Sequence (5′−3′)
VDR	Primer F	5′ GAATGTGCCTCGGATCTGTGG 3′
	Primer R	5′ ATGCGGCAATCTCCATTGAAG 3′
Wnt10b	Primer F	5′ CTCCACTACAGCCCAGAAC 3′
	Primer R	5′ CTCCCAAGAGCCTGACAAG 3′
β-catenin	Primer F	5′ TCACGCAAGAGCAAGTAG 3′
	Primer R	5′ CTGGACATTAGTGGGATGAG 3′
Osterix	Primer F	5′ TGCCTACTTACCCATCTGAC 3′
	Primer R	5′ TTGCCCACTATTGCCAAC 3′
Runx2	Primer F	5′ TTTGCCCTCATCCTTCAC 3′
	Primer R	5′ GCTTCTGCTACCACTCTAAC 3′
RANKL	Primer F	5′ ATGAAACTCACAGCCCTCTC 3′
	Primer R	5′ CATCGGAATACCTCTCCCAATC 3′
OPG	Primer F	5′ AGGGCATACTTCCTGTTG 3′
	Primer R	5′ TTCCTGGGTTGTCCATTC 3′
GADPH	Primer F	5′ ATCACTGCCACCCAGAAG 3′
	Primer R	5′ TCCACGACGGACACATTG 3′

### Metagenomic data processing and quality control

The metagenome sequencing was conducted utilizing the Illumina NovaSeq 6000 platform, provided by Beijing Nuohe Biomedical Technology Co., Ltd. Initial data refinement involved the application of Trimmomatic software to eliminate sequences of poor quality, retaining only those exceeding 60 bp in length with an average quality score above the threshold of 30 (21). Following this, the refined sequences were mapped to the human reference genome, version GRCh38, employing BWA, Samtools, and BEDTools to meticulously eliminate any sequences of human origin (22, 23).

### Statistical analysis

Statistical analysis of the data was conducted employing GraphPad Prism version 6 and SPSS software. For pairwise group comparisons, an independent-samples *t*-test was utilized, while one-way analysis of variance (ANOVA) was applied to identify differences among three or more groups. Statistical significance was based on the model group with a threshold of *P* < 0.05. Additionally, macrogenomic data analysis was performed with the aid of the psych and ggplot2 libraries within the R programming environment.

## RESULTS

### Effect on VD metabolites in fermentation supernatants

**Fig 1:**
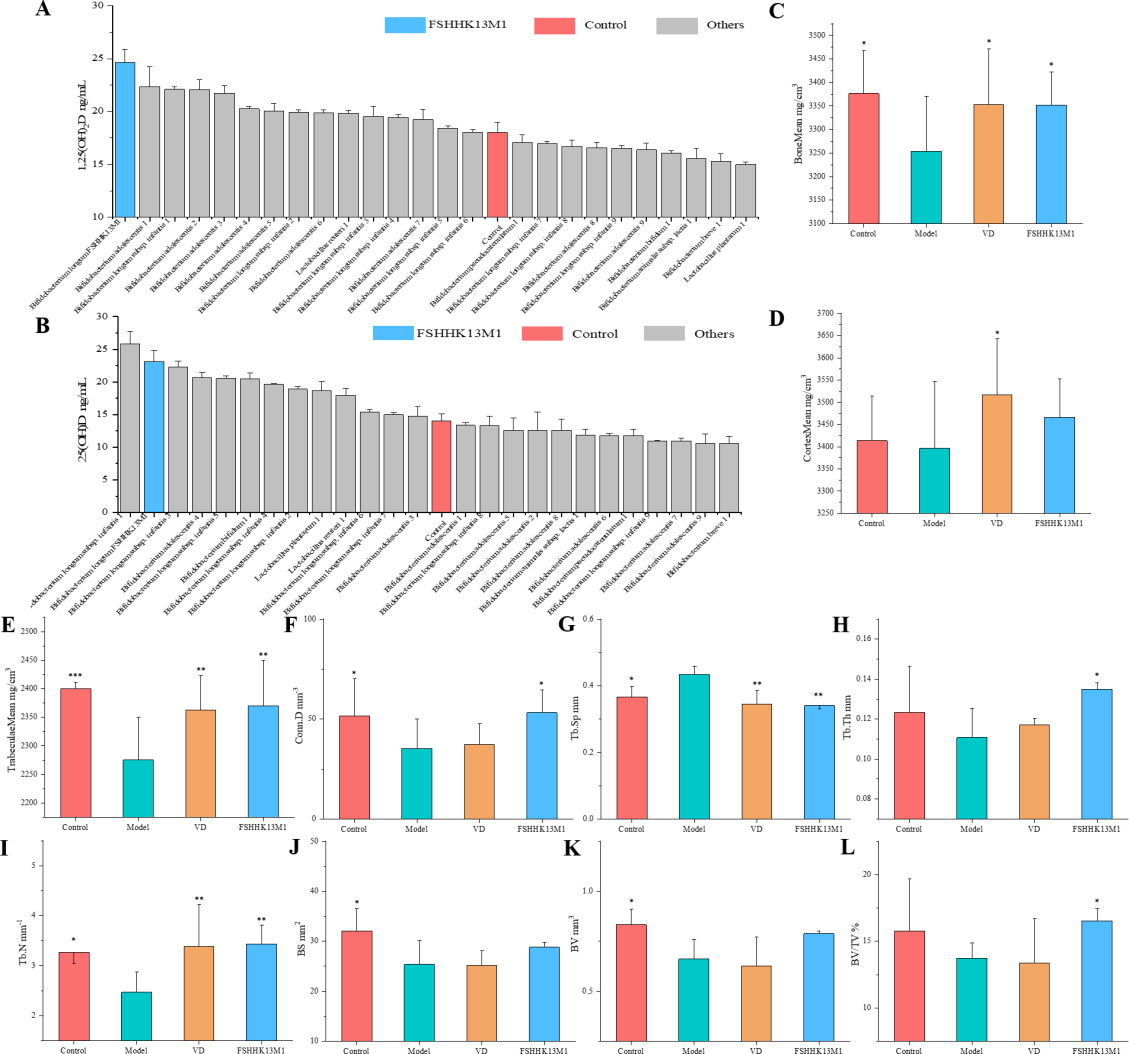
(**A**) 25(OH)D and (**B**) 1,25(OH)_2_D concentrations in fermentation supernatants. BMD of (**C**) the total, (**D**) cortical, and (**E**) trabecular bone of the distal femur; (**F**) Conn.D, (**G**) trabecular separation (Tb.Sp), (**H**) trabecular thickness (Tb.Th), and (**I**) number of bone trabeculae (Tb.N); (**J**) bone surface (BS), (**K**) BV, and (**L**) BV/TV were analyzed using micro-CT. Values are presented as the mean ± SEM (*n* = 6). * indicates significant differences from the model group: *P < 0.05，**P < 0.01，and ***P < 0.001. Groups: control, model, VD, and FSHHK13M1.

Twenty-five strains of 10 different bacterial species were selected for *in vitro* fecal fermentation to assess 1,25(OH)_2_D in the supernatant. The results showed that *B. longum* could increase the level of VD active forms more significantly at the species level. We chose the best functional performer, FSHHK13M1, for subsequent studies. Compared with the control group without bacterial inoculation, the addition of 0.1 mL of *B. longum* FSHHK13M1 to the *in vitro* fermentation model significantly increased the levels of the VD metabolites 25(OH)D and 1,25(OH)_2_D in the *in vitro* fermentation supernatant, as depicted in Fig. 1.

1,25(OH)_2_D is an agonist of the VDR, which, upon binding, regulates the transcription involved in skeletal health. It also serves as a therapeutic agent for osteoporosis. Therefore, *B. longum* FSHHK13M1, which demonstrated the highest ability to enhance 1,25(OH)_2_D levels, was selected to investigate its effect on RA-induced osteoporosis in mice.

### Effects on bone structures

Bone mineral density (BMD) serves as a crucial measure for the diagnosis of osteoporosis, providing insights into an individual’s bone metabolic status. Following a period of osteoporosis induction for 3 weeks, coupled with an additional 3 weeks of treatment with the test strain via gavage, the distal regions of the mice’s femurs were subjected to micro-CT analysis. The findings indicated a statistically significant reduction in both BM and TMC in the experimental group when compared with controls, as denoted by a *P*-value less than 0.05.

**Fig 2:**
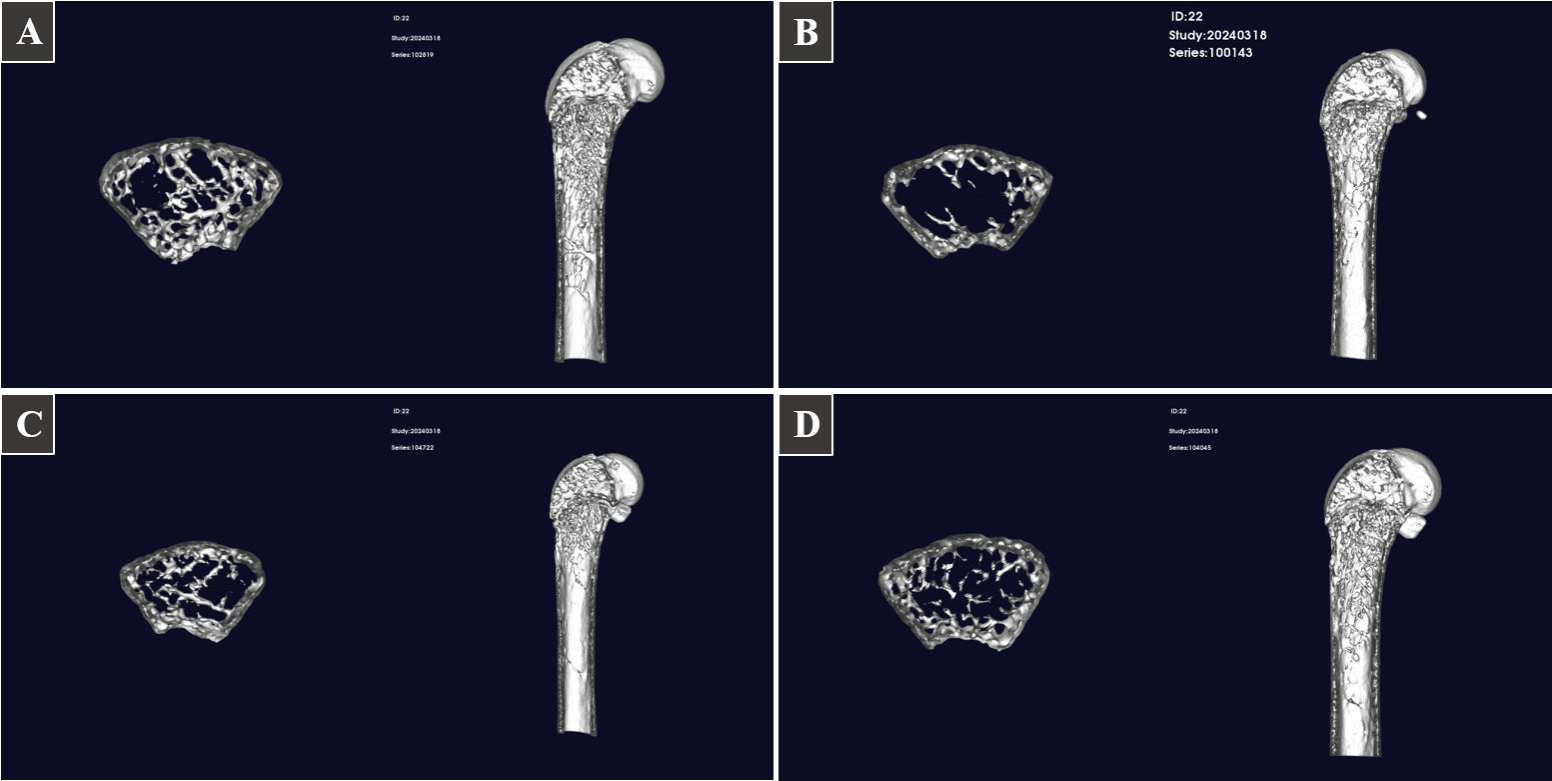
The three-dimensional reconstructions of mouse tibia. (A) control group; (B) model group; (C) VD group; (D) FSHHK13M1 group.

The results suggest a successful RA-induced osteoporosis model that did not exhibit spontaneous recovery within 3 weeks. Following 3 weeks of *B. longum* gavage treatment, both BM and TM content in mice saw a significant rise, reaching levels comparable to those in the vitamin D-supplemented group. Further analysis of bone microstructure, as depicted in Fig. 1, revealed that while Tb.Th and BV/TV in the model group were lower than the control, the differences were not statistically significant. Conversely, other parameters, such as Tb.N, Conn.D, BS, and BV showed a significant decrease, with an increase in Tb.Sp, all indicated significant changes at the *P* < 0.05 level. The BS and BV of mice increased after *B. longum* intervention, but the difference was not significant. Tb,Th, Tb.N, Conn.D, and BV/TV were significantly increased (*P* < 0.05), whereas Tb.Sp was significantly decreased (*P* < 0.05). VD intervention resulted in significant improvements only in the CM, TM, Tb.Sp, and Tb.N. These data suggest that RA causes the development of osteoporotic symptoms and decreases BMD in mice. *B. longum* FSHHK13M1 improved this condition with better results than VD supplementation.Meanwhile the 3D reconstruction of mouse tibia in Fig. 2 shows significantly that the bone trabeculae of osteoporotic mice improved after the intervention.

### Effects on serum bone metabolism markers

Serum levels of 25(OH)D serve as an indicator of the body’s vitamin D reserves. The metabolite 1,25(OH)2D is a crucial form of the vitamin, playing a vital role in maintaining calcium homeostasis. When administered orally, 1,25(OH)2D facilitates the normal absorption of calcium in the intestines, which helps to correct hypocalcemia and can either reduce or stabilize elevated levels of serum ALP. Increased levels of VD metabolites can reduce elevated plasma parathyroid concentrations, thereby promoting bone mineralization. Induced by RA, osteoporosis in mice led to a significant decrease in serum levels of both the storage form, 25(OH)D, and the active metabolite, 1,25(OH)2D, with the former showing a highly significant reduction (*P* < 0.001) and the latter a significant one (*P* < 0.05). This reduction in VD levels suggests a potential impairment in the vitamin’s physiological functions, the extent and mechanisms of which are not fully understood and may be compromised. After intervention with the gavage strain, the FSHHK13M1 group showed a significant increase in the serum VD metabolite 1,25(OH)_2_D level in osteoporotic mice compared with the model group (*P* < 0.01), and the 25(OH)D level increased but not significantly.

**Fig 3:**
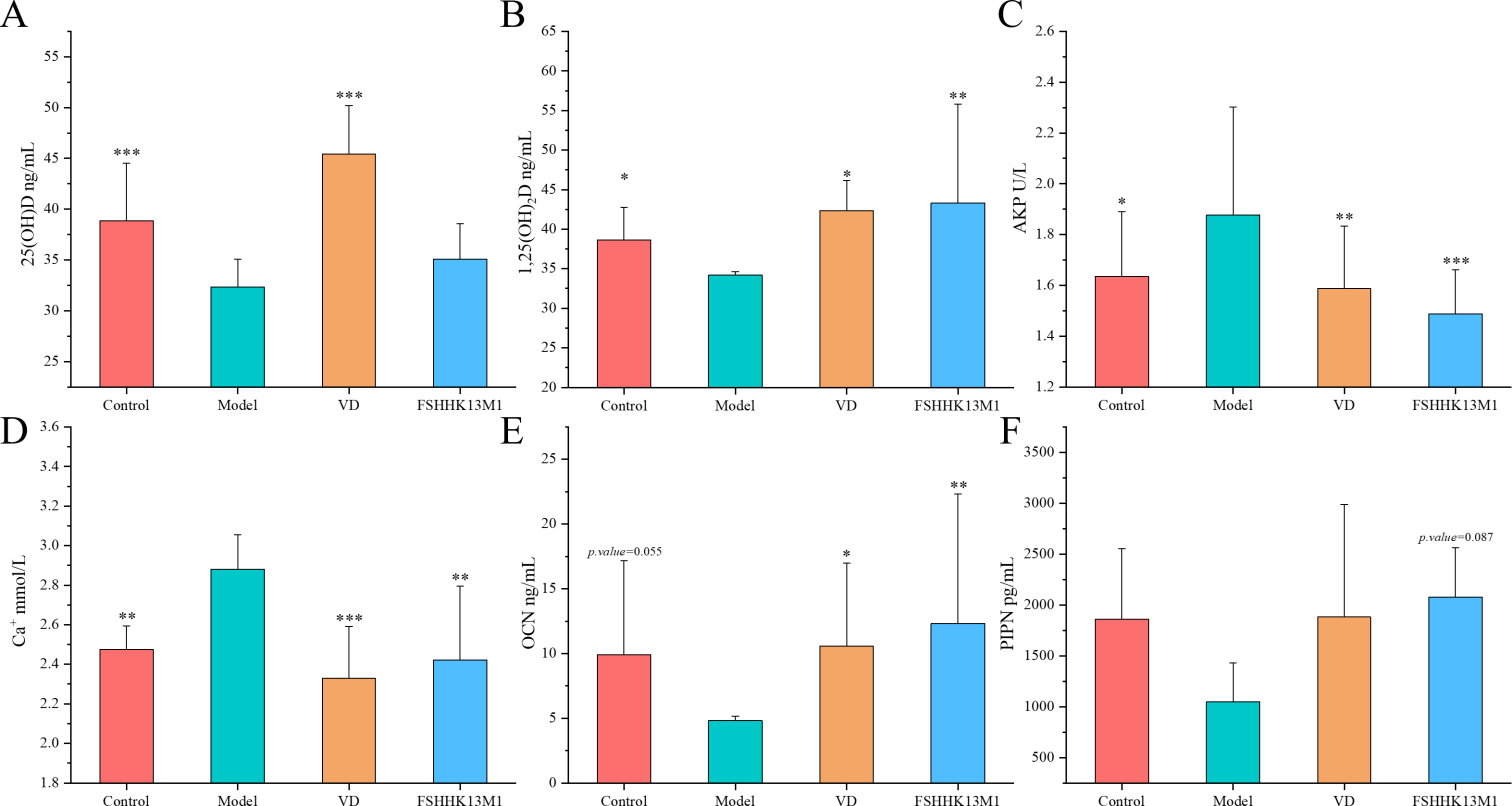
Effects on serum (**A**) 25(OH)D, (**B**) 1,25(OH)_2_D, (**C**) ALP, (**D**) Ca+, (**E**) OCN, and (**F**) PINP concentration in mice. * indicates significant differences from the model group: *P < 0.05, **P < 0.01, and ***P < 0.001. Groups: control, model, VD, and FSHHK13M1.

Abnormally excessive elevation of serum calcium ions may reflect bone calcium loss. ALP serves as an indicator of osteoblast metabolism, directly correlating with the activation of these bone-forming cells. OCN assays provide insights into the functionality of osteoblasts, while the level of PINP is indicative of new bone formation and the vitality of osteoblasts. Fig. 3 illustrates that RA-induced osteoporosis led to a statistically significant increase in both serum calcium and ALP levels in mice (*P* < 0.05), suggesting heightened osteoblast activity. This leads to bone calcium loss and bone damage in mice, culminating in the formation of an osteoporosis mouse model. After performing the intervention, serum calcium (*P* < 0.001) and ALP (*P* < 0.01) levels were significantly reduced in the VD group. Gavage of strain FSHHK13M1 significantly reduced serum calcium (*P* < 0.01) and ALP levels (*P* < 0.001) in osteoporotic mice. RA-induced osteoporosis reduced serum OCN and PINP levels in mice. This suggests that RA reduced the function and number of osteoblasts in mice. In comparison, the VD group exhibited significantly elevated serum OCN levels when juxtaposed with the model group, denoting a *P*-value of less than 0.05. Although serum levels of PINP were also higher in the VD group, this increase did not reach statistical significance. The gavage strain was also able to increase serum OCN (*P* < 0.01) and PINP levels in osteoporotic mice, which was better than treatment with VD.

These results suggested that RA-induced osteoporosis reduced serum active VD levels, promoted bone calcium loss, and inhibited osteoblastic activity. *B. longum* FSHHK13M1 had a better functional effect than VD supplementation alone, increasing VD metabolite levels, mitigating bone calcium loss, decreasing ALP levels, increasing bone production, and improving osteoblastic activity.

### Effects on mRNA expression of bone metabolism-related genes

Wnt10b/β-catenin signaling pathway regulates osteoblast proliferation, differentiation, and apoptosis and participates in bone cell metabolism through WNT protein. Wnt eventually causes inhibition of the phosphorylation process of β-catenin, so that β-catenin is enriched in the cell and translocated into the nucleus. In the nucleus, β-catenin binds to LEF1/TCF to initiate transcription and expression of target genes, Runx2 and Osterix, thereby regulating the proliferation and differentiation of osteoblasts. The OPG–RANK–RANKL signaling pathway primarily governs coupling between osteoblast and osteoclast activity. When osteoporosis occurs, mRNA expression levels of RANKL is upregulated in bone marrow cells, resulting in reduced osteocalcin synthesis in bone cells and accelerated bone resorption. OPG inhibits RANKL-induced osteoclast differentiation and maturation by competing with RANK to bind to RANKL, so the ratio of RANKL/OPG plays a decisive role in the proliferation and differentiation of osteoclasts. Researchers have confirmed that the OPG–RANK–RANKL signaling pathway and the Wnt/β-catenin signaling pathways are interrelated (24). Under the regulation of these major signaling pathways, osteoblasts and osteoclasts together determine bone volume and bone remodeling.

**Fig 4:**
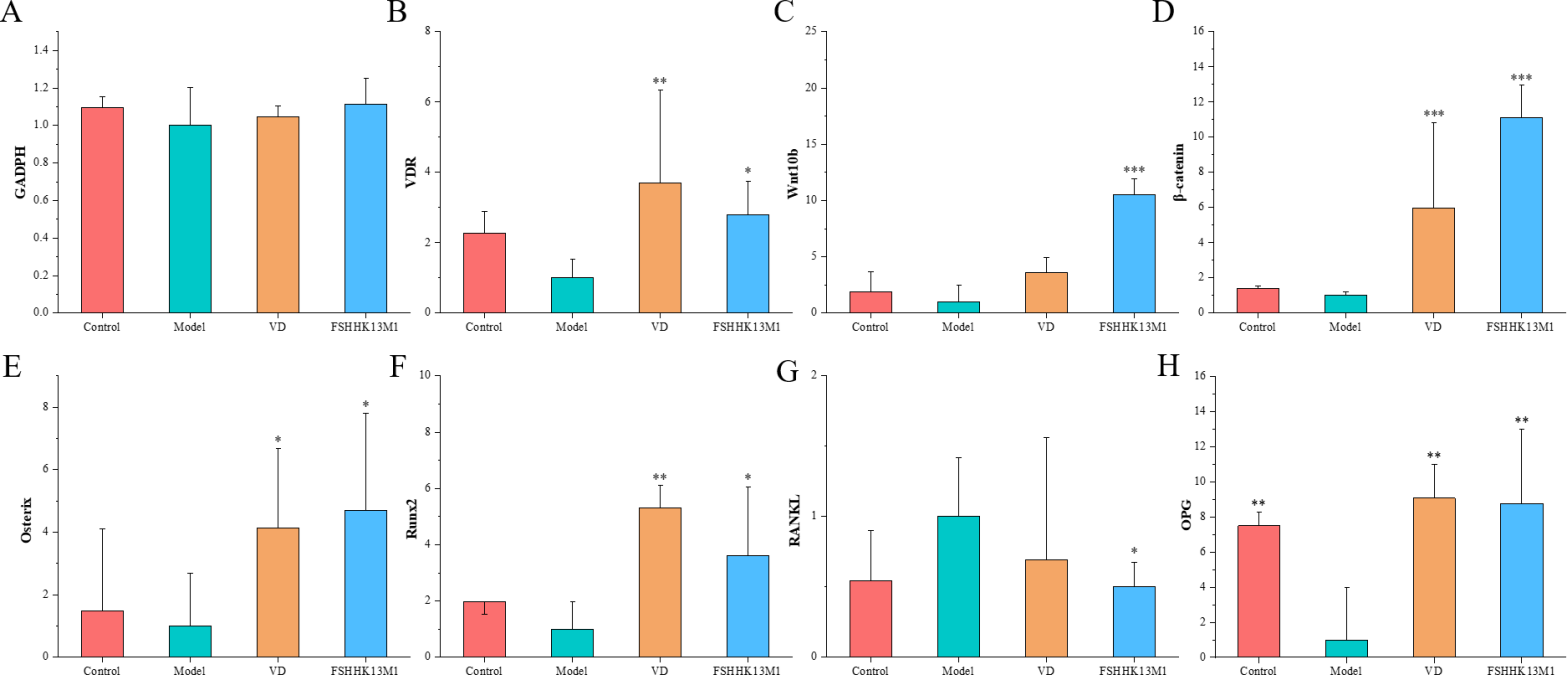
mRNA expression of bone metabolism related genes in mice. (**A**) GADPH, (**B**) VDR, (**C**) Wnt10b, (**D**) β-catenin, (**E**) Osterix, (**F**) Runx2, (**G**) RANKL, and (**H**) OPG. * indicates significant differences from the model group: *P < 0.05, **P < 0.01, and ***P < 0.001. Groups: control, model, VD, and FSHHK13M1.

We targeted bone-related gene expression in osteoblasts and osteoclasts to reflect their function. These genes included the vitamin D receptor, Wnt10b/β-catenin pathway, Runx2/Osterix pathway, and OPG/RANKL/RANK pathway. The mechanism of using retinoic acid to prepare osteoporosis model is that retinoic acid stimulates both osteoclasts and osteoblasts, leading to a high rate of bone transformation, making bone absorption exceed bone formation, and finally leading to osteoporosis. As shown in Fig. 4, this is consistent with our results. Meanwhile, compared with the control group, the mRNA expression levels of RNAKL in the model group were significantly increased. However, the mRNA expression levels of OPG were significantly decreased. Thus, the ratios of RANKL/OPG were significantly increased. We found that vitamin D and *B. longum* FSHHK13M1 activated the expression of the VDR, Wnt10b/β-catenin, and Runx2/Osterix pathways to increase the level of osteoblast function and stimulate osteoblast differentiation. They significantly increase the expression of OPG, inhibit the expression of RANKL/RANK pathway, and inhibit the differentiation and activation of osteoclasts.

### Effects on the diversity of gut microbiota

**Fig 5:**
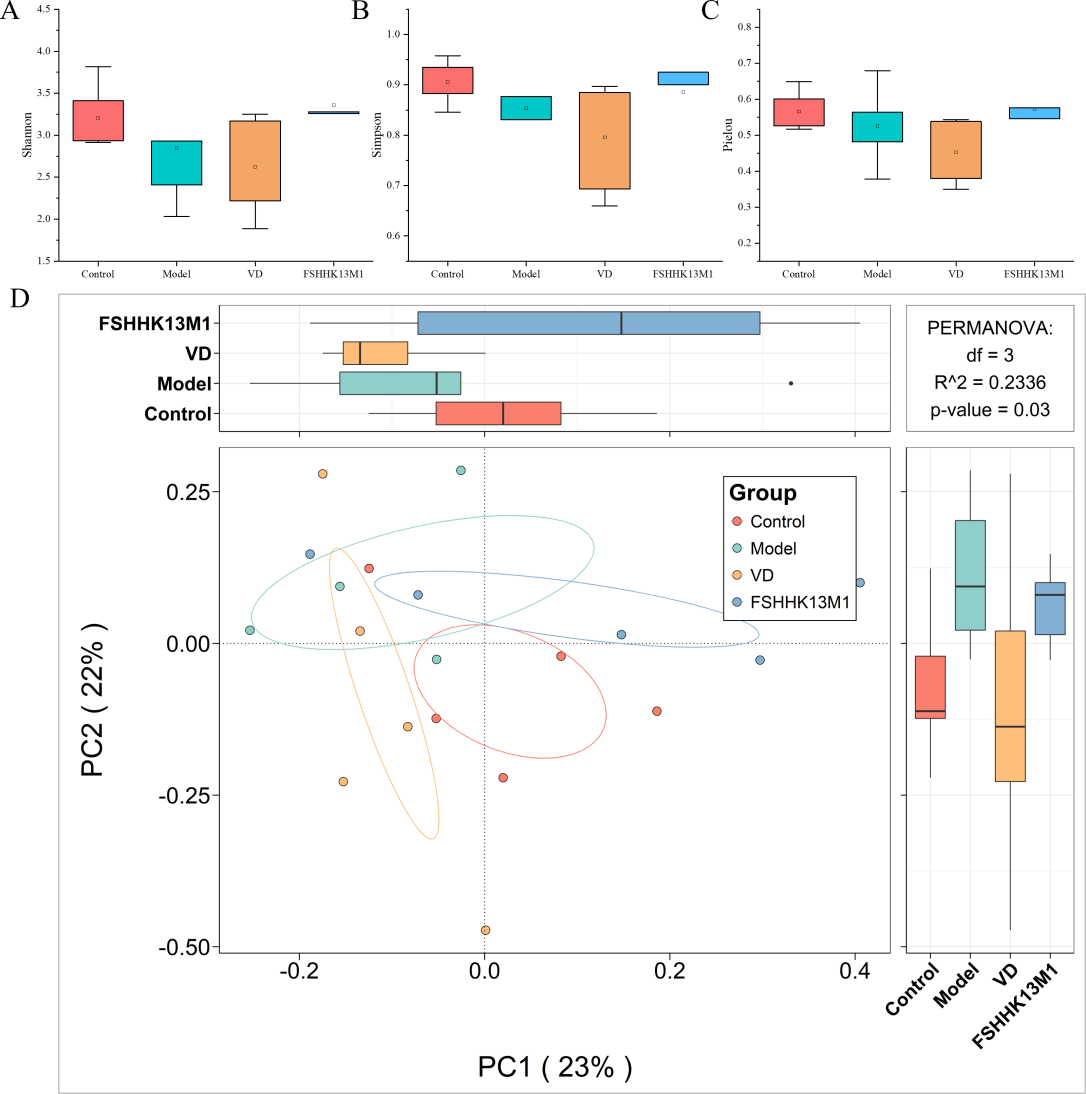
Species diversity analysis of gut microbiota in the different groups. (**A**) Shannon index, (**B**) Simpson index, (**C**) Pielou index, and (**D**) β-diversity based on the PCoA algorithm. Groups: control, model, VD, and FSHHK13M1.

Research has consistently demonstrated the capacity of probiotics to modulate gut microbiota composition. Diversity indices, including Pielou’s homogeneity, Shannon, and Simpson, for various groups are depicted in Fig. 5. In the model group, the indices indicated a reduction in gut microbiota species diversity and a decrease in both species distribution uniformity and overall diversity when compared with the control group. However, these variations did not attain statistical significance, suggesting that while there were observable trends, they were not conclusively different.

Following the administration of VD and FSHHK13M1 via gavage, no noteworthy alterations were observed in the Pielou evenness, Shannon, or Simpson diversity indices of the murine gut microbiota. The lack of significant divergence in these indices between the experimental groups treated with VD and FSHHK13M1 and the control group could be attributed to a potential rise in the prevalence of certain advantageous microbial species. Such a selective enrichment might alter the composition of the gut microbiota, potentially causing a proportional reduction in the presence of other, non-targeted microorganisms. This dynamic balance could culminate in an overall equilibrium in species count within the microbial community.

Principal coordinate analysis (PCoA), grounded in Bray–Curtis distance metrics, facilitates a visual examination of gut microbiota species diversity among individuals, highlighting both differences and similarities in their microbial profiles. The PCoA revealed that the gut microbiota configuration in osteoporotic mice markedly diverged from that of healthy counterparts. Notably, the microbiota of mice in both the VD and FSHHK13M1 groups exhibited statistically distinct species compositions compared with the model group. These findings suggest that the intervention with *B. longum* effectively restructured the gut microbiota in RA-induced osteoporotic mice.

### Effects on the structural composition of gut microbiota

**Fig 6:**
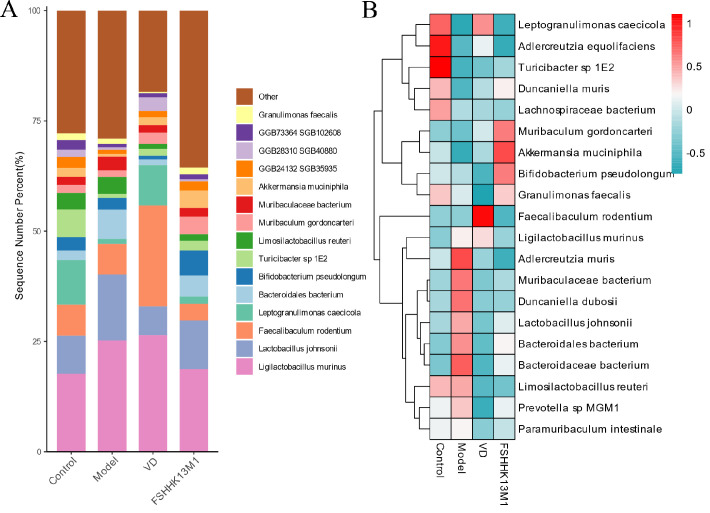
(**A**) Species-level relative abundance stacked plot, (**B**) species-level heatmap of grouped clustering. Groups: control, model, VD, and FSHHK13M1.

To further investigate changes in gut microbiota composition, community characteristics at the species level were analyzed and compared. The stacked plots of relative abundance for the top 12 species ranked based on relative abundance further illustrate the differences in species abundance between the groups. As depicted in Fig. 6, *Ligilactobacillus murinus* was the dominant species in the gut microbiota of all four groups of mice. The relative abundances of *L. murinus* and *Bacteroidales bacterium* were significantly reduced after VD and *B. longum* FSHHK13M1 interventions and were restored to a structure similar to that of the model group. After VD intervention, the relative abundance of *F. rodentium* and *Leptogranulimonas caecicola* significantly increased. *B. pseudolongum*, *Muribaculum gordoncarteri,* and *A. muciniphila* significantly increased after FSHHK13M1 intervention.

The hierarchical clustering heatmap, which depicted the 20 most prevalent species based on their relative abundance, revealed that the administration of *B. longum* FSHHK13M1 led to a marked elevation in the relative proportions of *B. pseudolongum*, *A. muciniphila*, and *M. gordoncarteri*. Furthermore, following the introduction of VD, there was a notable surge in the prevalence of *F. rodentium* within the gut microbiota.

The grouped clustering heatmap of the top 20 species in terms of relative abundance showed that *B. longum* FSHHK13M1 significantly increased the relative abundances of *B. pseudolongum*, *A. muciniphila*, and *M. gordoncarteri*. After the VD intervention, the relative abundance of *F. rodentium* significantly increased.

**Fig 7:**
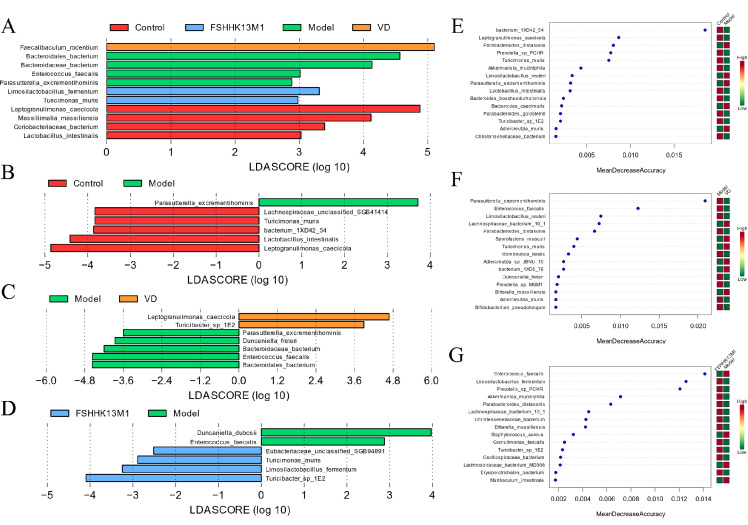
LEfSe analysis of fecal microbiota metagenomic species in mice: (**A**) All groups, (**B**) control vs model, (**C**) model vs VD, and (**D**) model vs FSHHK13M1. Species-level heatmap of grouped clustering. Importance analysis of key species characteristics based on RF model: (**E**) control vs model, (**F**) model vs VD, and (**G**) model vs FSHHK13M1.

The impact of VD and *B. longum* FSHHK13M1 on the gut microbiota of osteoporotic mice was scrutinized through linear discriminant analysis effect size (LEfSe) and random forest (RF) analyses. As depicted in Fig. 7, significant alterations in the relative abundance of specific microbes were observed. Notably, *F. rodentium* in the VD group and *L. fermentum* in the FSHHK13M1 group exhibited a marked increase, both reaching a logarithmic fold change (LDA) score greater than 3, with statistical significance (*P* < 0.05). Additionally, a two-by-two comparison revealed a significant increase in the relative abundance of *Parasutterella excrementihominis* in the model group, with an LDA score exceeding 2, indicative of statistical significance (*P* < 0.05). Collectively, these findings indicate that both VD and FSHHK13M1 interventions may promote the growth of beneficial bacteria while curbing the proliferation of others, thereby potentially restoring the gut microbiota balance disrupted in osteoporotic mice. This modulation of the gut microbiota may contribute to mitigating osteoporotic symptoms.

### Correlation analysis between gut microbiota and physiological and biochemical indicators

**Fig 8:**
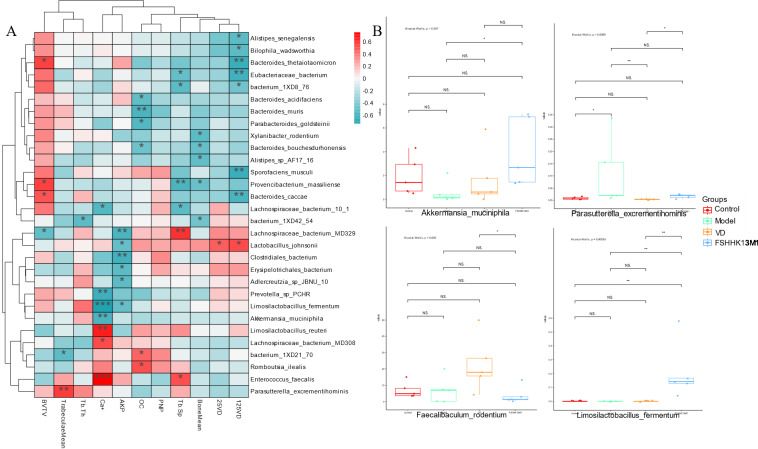
(**A**) Spearman correlation analysis of the relative abundance of gut microbial species with serum levels of 25(OH)D, 1,25(OH)2D, BM, TM, Tb.Th, Tb.Sp, BV/TV, Ca+, ALP, OCN, and PINP in the different groups of mice; (**B**) Wilcoxon rank-sum test analysis of differential species relative abundance in mouse gut microbiota among different groups. * indicates significant differences from the model group: *P < 0.05，**P < 0.01，and ***P < 0.001. Groups: control, model, VD, and FSHHK13M1.

The relative abundance of intestinal flora was correlated with skeletal health indicators in mice, as shown in Fig. 8; *Lactobacillus johnsonii* and *L. fermentum* are positively correlated with most of the beneficial indicators and negatively correlated with calcium loss and bone trabecular gap. *A. muciniphila* is negatively correlated with calcium loss and trabecular gaps. This is consistent with a previously reported relationship between a significant increase in the relative abundance of *A. muciniphila* after FSHHK13M1 intervention and the alleviating effect of FSHHK13M1 on osteoporosis. Most *Bacteroides* were negatively correlated with the levels of VD metabolites and beneficial indicators, corresponding to a significant reduction in the relative abundance of *B. bacterium* after VD and FSHHK13M1 interventions. This result was corroborated by Wilcoxon rank-sum test analysis, in which VD intervention significantly increased the relative abundance of *F. rodentium*, whereas FSHHK13M1 intervention increased the relative abundance of *A. muciniphila,* and *L. fermentum* significantly increased their relative abundance after FSHHK13M1 intervention. These results may explain the role of VD and *B. longum* FSHHK13M1 in terms of the intestinal flora, which exerts its mitigating effect on osteoporosis through the regulation of specific intestinal flora.

## DISCUSSION

RA is a derivative of vitamin A, which inhibits bone formation and promotes bone resorption. The pathological process of osteoporosis can be mimicked in mice by administering retinoic acid, which results in decreased bone density and altered bone organization. This model has been widely used in several studies, owing to its high reproducibility and stability. When the serum calcium and ALP levels of the mice in the model group were significantly higher than those of the blank group, it was demonstrated that bone loss was severe, and the osteoporosis model was successful. Abnormally high serum calcium levels indicate decreased bone calcium storage capacity, severe calcium loss, poor bone health, increased bone metabolism, and osteoporosis. Bone diseases, such as rickets, chondromalacia, bone malignancies, and bone metastases from malignant tumors, can lead to abnormally elevated ALP levels. OCN, also known as bone hydroxyglutamic acid protein (BGP), is a hormone-like peptide produced and secreted by osteoblasts. Serum OCN is a biochemical marker of the osteoblast function. Approximately 20% of the OCN synthesized by osteoblasts is released into the blood, and there is a positive correlation between serum OCN and bone tissue OCN. Therefore, blood OCN measurements can reflect the functional status of osteoblasts. The expression level of PINP reflects the formation of new bone. When osteoblast synthesis is reduced, the level of PINP, which reflects changes in newly synthesized type I collagen, decreases.

In this study, a strain of *B. longum* FSHHK13M1, was screened using an *in vitro* fermentation model. It increases the levels of VD metabolites and alleviates osteoporosis symptoms. An osteoporotic mouse model was established by gavage RA. The ameliorative effect of *B. longum* FSHHK13M1 on osteoporosis symptoms in mice was investigated in terms of bone structural indices, such as BMD, Tb.Sp, and BV/TV, and serum biochemical indices, such as serum OCN, PINP, ALP, and serum calcium. Moreover, *B. longum* FSHHK13M1 significantly increased serum levels of the VD metabolites 25 (OH)D and 1,25 (OH)_2_D in mice. These results indicate that *B. longum* FSHHK13M1 could regulate bone metabolism in osteoporotic mice and significantly improve RA-induced osteoporosis symptoms in mice.

We believe that the main reasons for the alleviating effect of *B. longum* FSHHK13M1 on RA-induced osteoporosis in mice are as follows. First, the VD plays an important role in bone and calcium absorption, primarily by helping humans to absorb calcium from the gut into the bloodstream. It also helps to move calcium from the blood to the bones. In particular, in calcium-deficient children and middle-aged and elderly patients, it has a better effect and can help osteoblasts in the calcium not to have an excessive loss to avoid the occurrence of osteoporosis and bone growth and development disorders in children (25, 26). Several studies have shown that the gut microbiota alters the metabolism of VD in the gut and that probiotic supplements can influence circulating VD levels (18). Our research highlights several critical clinical implications. Extensive epidemiological evidence has linked low serum levels of VD to an increased risk of a spectrum of adverse health outcomes. These include skeletal disorders, such as osteoporosis, metabolic disorders, such as obesity and diabetes, inflammatory disorders, including inflammatory bowel disease, and major diseases, such as cardiovascular disease, cancer, and autoimmune diseases (27–29). The association suggests that maintaining adequate vitamin D levels may be critical in preventing a range of health complications.

However, the largest randomized clinical trial to date involving 25,000 adults showed that VD supplementation had no significant effect on health outcomes, including heart disease, cancer, fall risk, and bone health (30–33). Studies have found that individuals with different levels of response to VD3 supplements are distinguished as high, medium, or low responders. Approximately 25% of the interviewed group showed fewer responders in terms of VD target gene regulation of expression (34–37). This finding implies that low responders require higher daily doses of VD3 supplements than those recommended by population-based recommendations and guidelines. The ability of the body to metabolize VD into its active form is as important as VD supplementation (38).

*B. longum* FSHHK13M1 increases the levels of vitamin D metabolites 25(OH)D and 1,25(OH)_2_D. Clinically, the indicator for assessing VD deficiency is the blood level of 25(OH)D, including 25-hydroxy VD_3_ and 25-hydroxy VD_2_ (38–40). The activation rate of VD is determined by the ratio of 1,25(OH)_2_D to 25(OH)D and can be used to calculate the metabolic flux and activation level of VD. Clinically, the level of 1,25(OH)_2_D is measured to determine the level of active VD in metabolic bone diseases. Specific gut microorganisms in the human body can metabolize VD (41), and blood levels of 1,25(OH)_2_D, the active form of VD, are correlated with specific gut microorganisms such as *Blautia* and *Ruminococcus* (42). Therefore, the gut microbiota may determine the bioavailability of VD. These studies suggest that gut microbiota plays an important role in the bioavailability of VD in humans. It has been suggested that the gut microbiota can be regulated in patients to improve the physiological activity of the VD to maintain bone health.

The VD active form, 1,25(OH)_2_D, is a direct agonist of VDR, which is widely present in various organs in the body, giving 1,25(OH)_2_D a multifaceted and important role (43). When the VD metabolite 1,25(OH)_2_D enters the cell, it binds to VDR, forming a 1,25(OH)_2_D–VDR complex (44). This complex can enter the nucleus and bind to the vitamin D response element (VDRE), which regulates the transcription of a range of genes (44). The 1,25(OH)_2_D–VDR complex can activate several genes, such as NFE2L2, to alleviate oxidative stress (45), calcium channel genes (such as TRPV6) (46), calcium-binding protein genes (such as calbindin-D28k), parathyroid hormone (PTH), and fibroblast growth factor 23 (FGF23) (47). Activation of these genes promotes the intestinal absorption of calcium and phosphorus, increases the availability of calcium and phosphorus in the body, increases active VD levels, and contributes to bone health. The 1,25(OH)_2_D–VDR complex can also activate several bone formation-related genes, such as OCN and osteopontin (48). Activation of these genes promotes the activity of bone-forming cells and the synthesis of the bone matrix, thereby increasing the density and strength of bones. Also, the 1,25(OH)_2_D–VDR complex inhibits the expression of bone resorption-related genes, such as the gene for osteoclast differentiation factor (RANKL) and the gene for osteoclastogenic hormone (M-CSF) (49). Suppression of these genes reduces osteoclast formation and activation, thereby decreasing bone destruction in the skeleton. The 1,25(OH)_2_D–VDR complex also regulates the expression of apoptosis and inflammation-related genes, such as Bcl-2 and NF-κB (50). The anti-inflammatory effects of 1,25(OH)_2_D may help reduce inflammatory damage to the bone and minimize the development of osteoporosis symptoms.

In addition, VD and *B. longum* FSHHK13M1 significantly increased the relative abundances of *A. muciniphila*, *L. fermentum*, *B. pseudolongum*, and *F. rodentium. A. muciniphila* has repeatedly been reported to be associated with the gut barrier (51). *L. fermentum* and *B. pseudolongum* are common beneficial flora that have been reported to slow intestinal inflammatory responses in mice (52, 53). This was chosen as a mouse model of RA-induced osteoporosis, considering that RA causes some damage and pro-inflammatory effects in the intestine, which may affect the regulation of VD and calcium absorption or VDR expression. *A. muciniphila*, *L. fermentum*, and *B. pseudolongum* may play important roles in this regard. *F. rodentium*, on the other hand, remodels retinoic acid signaling to control eosinophil-dependent intestinal epithelial homeostasis, again playing a role in the intestinal barrier and homeostasis (54).

1,25(OH)_2_D is not only an important active VD for maintaining bone health but has also recently been found to be important for cell proliferation, differentiation, and the immune system (55). 1,25(OH)_2_D is also an important hormone for immune regulation. In the same way that VDR is expressed at multiple sites throughout the body, the enzyme 1-alpha hydroxylase (CYP27B1), which activates VD and produces 1,25(OH)_2_D, has also been found at multiple sites, in addition to the kidneys. Calcitriol has been shown to be produced by the presence of 1α-hydroxylase in the placenta, monocytes, macrophages, tumor cell supernatants, lymph nodes of patients with nodal disease, and keratinocytes (43, 56). Similarly, CYP27B1 expression was detected in colon epithelial cells (57). The multiple roles of 1,25(OH)_2_D in bone health include immunomodulation, cellular differentiation, CYP27B1 expression, and VDR expression in the colon. There may be a deeper link between intestinal homeostasis and the barrier and physiologically active functions of the VD. This may explain how *B. longum* FSHHK13M1 increases VD metabolite levels in the gut by influencing the gut microbiota. This may also explain why the key differential microbiota that were altered after *B. longum* FSHHK13M1 intervention were associated with gut barriers and homeostasis. These results provide novel insights into how probiotics affect VD metabolite levels by regulating the gut microbiota to maintain bone health and gut homeostasis.

### Conclusion

In summary, we screened a strain of *B. longum* with the functional potential to enhance the physiological activity of VD using an *in vitro* fecal fermentation model. We also evaluated its effects on skeletal health in mice with RA-induced osteoporosis. After 3 weeks of RA gavage, the mice showed significant bone loss and reduced serum metabolite levels. After 3 weeks of continuous gavage of *B. longum* FSHHK13M1, there were significant improvements in BMD and Tb.N, improvement of serum calcium, reduction of ALP, enhancement of osteoblast activity, increase in VD metabolite levels, and modulation of the gut microbiota. We found that vitamin D and *B. longum* FSHHK13M1 activated the expression of the VDR, Wnt10b/β-catenin, and Runx2/Osterix pathways to increase the level of osteoblast function and stimulates osteoblast differentiation. They significantly increase the expression of OPG, inhibit the expression of RANKL/RANK pathway, and inhibit the differentiation and activation of osteoclasts. Using VD as a positive control, *B. longum* FSHHK13M1 showed greater improvement in osteoporosis. In addition, we analyzed the effect of *B. longum* on the gut microbiota of mice. We found that *B. longum* intervention with the VD significantly increased the relative abundances of *A. muciniphila*, *L. fermentum*, *B. pseudolongum*, and *F. rodentium*. These bacteria are closely associated with estramustine production, intestinal barrier function, and gut microbiota homeostasis. Considering the multiple roles of 1,25(OH)_2_D in bone health, immunomodulation, cellular differentiation, and the expression of CYP27B1 and VDR at the colonic site, the intestinal barrier and the physiologically active function of VD may be linked at a deeper level. Flora with increased relative abundance may be a target for further analysis to improve the physiological activity of VD. It may also possess the ability to increase VD physiological activity and metabolite levels; however, further experiments are needed to test this hypothesis.

## Data Availability

The raw sequencing data generated from this study have been deposited in NCBI SRA (https://www.ncbi.nlm.nih.gov/sra) under the accession number SRA: SRP553168 and BioProject: PRJNA1201336.

## References

[1] LeBoff MS, Greenspan SL, Insogna KL, Lewiecki EM, Saag KG, Singer AJ, Siris ES. 2022. The clinician’s guide to prevention and treatment of osteoporosis. Osteoporos Int 33:2049–2102. doi:10.1007/s00198-021-05900-y 35478046 PMC9546973

[2] Khan AA, Morrison A, Hanley DA, Felsenberg D, McCauley LK, O’Ryan F, Reid IR, Ruggiero SL, Taguchi A, Tetradis S, et al.. 2015. Diagnosis and management of osteonecrosis of the jaw: a systematic review and international consensus. J Bone Miner Res 30:3–23. doi:10.1002/jbmr.2405 25414052

[3] Patel DV, Bolland M, Nisa Z, Al-Abuwsi F, Singh M, Horne A, Reid IR, McGhee CNJ. 2015. Incidence of ocular side effects with intravenous zoledronate: secondary analysis of a randomized controlled trial. Osteoporos Int 26:499–503. doi:10.1007/s00198-014-2872-5 25187119

[4] Shoback D, Rosen CJ, Black DM, Cheung AM, Murad MH, Eastell R. 2020. Pharmacological management of osteoporosis in postmenopausal women: an endocrine society guideline update. J Clin Endocrinol Metab 105:587–594. doi:10.1210/clinem/dgaa048 32068863

[5] Crandall CJ, Hovey KM, Andrews C, Cauley JA, Stefanick M, Shufelt C, Prentice RL, Kaunitz AM, Eaton C, Wactawski-Wende J, Manson JE. 2017. Comparison of clinical outcomes among users of oral and transdermal estrogen therapy in the Women’s Health Initiative Observational Study. Menopause 24:1145–1153. doi:10.1097/GME.0000000000000899 28697036 PMC5607093

[6] Overman RA, Borse M, Gourlay ML. 2013. Salmon calcitonin use and associated cancer risk. Ann Pharmacother 47:1675–1684. doi:10.1177/1060028013509233 24259626

[7] Roerholt C, Eiken P, Abrahamsen B. 2009. Initiation of anti-osteoporotic therapy in patients with recent fractures: a nationwide analysis of prescription rates and persistence. Osteoporos Int 20:299–307. doi:10.1007/s00198-008-0651-x 18551241

[8] Liu SK, Munson JC, Bell J-E, Zaha RL, Mecchella JN, Tosteson ANA, Morden NE. 2013. Quality of osteoporosis care of older Medicare recipients with fragility fractures: 2006 to 2010. J Am Geriatr Soc 61:1855–1862. doi:10.1111/jgs.12507 24219186 PMC4084674

[9] Kim SC, Kim M-S, Sanfélix-Gimeno G, Song HJ, Liu J, Hurtado I, Peiró S, Lee J, Choi N-K, Park B-J, Avorn J. 2015. Use of osteoporosis medications after hospitalization for hip fracture: a cross-national study. Am J Med 128:519–526. doi:10.1016/j.amjmed.2015.01.014 25660252 PMC4414898

[10] Bouillon R, Manousaki D, Rosen C, Trajanoska K, Rivadeneira F, Richards JB. 2022. The health effects of vitamin D supplementation: evidence from human studies. Nat Rev Endocrinol 18:96–110. doi:10.1038/s41574-021-00593-z 34815552 PMC8609267

[11] Harrison SR, Li D, Jeffery LE, Raza K, Hewison M. 2020. Vitamin D, autoimmune disease and rheumatoid arthritis. Calcif Tissue Int 106:58–75. doi:10.1007/s00223-019-00577-2 31286174 PMC6960236

[12] Latic N, Erben RG. 2020. Vitamin D and cardiovascular disease, with emphasis on hypertension, atherosclerosis, and heart failure. Int J Mol Sci 21:6483–6483. doi:10.3390/ijms21186483 32899880 PMC7555466

[13] Hanel A, Carlberg C. 2022. Time-resolved gene expression analysis monitors the regulation of inflammatory mediators and attenuation of adaptive immune response by vitamin D. Int J Mol Sci 23:911–911. doi:10.3390/ijms23020911 35055093 PMC8776203

[14] Cheng JB, Levine MA, Bell NH, Mangelsdorf DJ, Russell DW. 2004. Genetic evidence that the human CYP2R1 enzyme is a key vitamin D 25-hydroxylase. Proc Natl Acad Sci U S A 101:7711–7715. doi:10.1073/pnas.0402490101 15128933 PMC419671

[15] Ginsberg C, Ix JH. 2024. New insights into vitamin D metabolism in kidney disease and transplant. Am J Kidney Dis 84:400–402. doi:10.1053/j.ajkd.2024.06.003 39046404

[16] Latic N, Erben RG. 2021. FGF23 and vitamin D metabolism. JBMR Plus 5:e10558–e10558. doi:10.1002/jbm4.10558 34950827 PMC8674776

[17] Demay MB, Pittas AG, Bikle DD, Diab DL, Kiely ME, Lazaretti-Castro M, Lips P, Mitchell DM, Murad MH, Powers S, Rao SD, Scragg R, Tayek JA, Valent AM, Walsh JME, McCartney CR. 2024. Vitamin D for the prevention of disease: an endocrine society clinical practice guideline. J Clin Endocrinol Metab 109:1907–1947. doi:10.1210/clinem/dgae290 38828931

[18] Barbáchano A, Fernández-Barral A, Ferrer-Mayorga G, Costales-Carrera A, Larriba MJ, Muñoz A. 2017. The endocrine vitamin D system in the gut. Mol Cell Endocrinol 453:79–87. doi:10.1016/j.mce.2016.11.028 27913273

[19] Jones ML, Martoni CJ, Prakash S. 2013. Oral supplementation with probiotic L. reuteri NCIMB 30242 increases mean circulating 25-hydroxyvitamin D: a post hoc analysis of a randomized controlled trial. J Clin Endocrinol Metab 98:2944–2951. doi:10.1210/jc.2012-4262 23609838

[20] Yu J, Cao G, Yuan S, Luo C, Yu J, Cai M. 2021. Probiotic supplements and bone health in postmenopausal women: a meta-analysis of randomised controlled trials. BMJ Open 11:e041393. doi:10.1136/bmjopen-2020-041393 PMC7929795 33653743

[21] Bolger AM, Lohse M, Usadel B. 2014. Trimmomatic: a flexible trimmer for Illumina sequence data. Bioinformatics 30:2114–2120. doi:10.1093/bioinformatics/btu170 24695404 PMC4103590

[22] Li H. 2013. Aligning sequence reads, clone sequences and assembly contigs with BWA-MEM. Arxiv. doi:10.48550/arXiv.1303.3997

[23] Quinlan AR, Hall IM. 2010. BEDTools: a flexible suite of utilities for comparing genomic features. Bioinformatics 26:841–842. doi:10.1093/bioinformatics/btq033 20110278 PMC2832824

[24] Glass DA 2nd, Bialek P, Ahn JD, Starbuck M, Patel MS, Clevers H, Taketo MM, Long F, McMahon AP, Lang RA, Karsenty G. 2005. Canonical Wnt signaling in differentiated osteoblasts controls osteoclast differentiation. Dev Cell 8:751–764. doi:10.1016/j.devcel.2005.02.017 15866165

[25] Weaver CM, Gordon CM, Janz KF, Kalkwarf HJ, Lappe JM, Lewis R, O’Karma M, Wallace TC, Zemel BS. 2016. The National Osteoporosis Foundation’s position statement on peak bone mass development and lifestyle factors: a systematic review and implementation recommendations. Osteoporos Int 27:1281–1386. doi:10.1007/s00198-015-3440-3 26856587 PMC4791473

[26] Zhao J-G, Zeng X-T, Wang J, Liu L. 2017. Association between calcium or vitamin D supplementation and fracture incidence in community-dwelling older adults: a systematic review and meta-analysis. JAMA 318:2466–2482. doi:10.1001/jama.2017.19344 29279934 PMC5820727

[27] Bikle DD. 2016. Extraskeletal actions of vitamin D. Ann N Y Acad Sci 1376:29–52. doi:10.1111/nyas.13219 27649525 PMC5031366

[28] Gaksch M, Jorde R, Grimnes G, Joakimsen R, Schirmer H, Wilsgaard T, Mathiesen EB, Njølstad I, Løchen M-L, März W, et al.. 2017. Vitamin D and mortality: Individual participant data meta-analysis of standardized 25-hydroxyvitamin D in 26916 individuals from a European consortium. PLoS One 12:e0170791. doi:10.1371/journal.pone.0170791 28207791 PMC5312926

[29] Fan X, Wang J, Song M, Giovannucci EL, Ma H, Jin G, Hu Z, Shen H, Hang D. 2020. Vitamin D status and risk of all-cause and cause-specific mortality in a large cohort: results from the UK Biobank. J Clin Endocrinol Metab 105:e3606–e3619. doi:10.1210/clinem/dgaa432 32620963

[30] Donlon CM, LeBoff MS, Chou SH, Cook NR, Copeland T, Buring JE, Bubes V, Kotler G, Manson JE. 2018. Baseline characteristics of participants in the VITamin D and OmegA-3 TriaL (VITAL): effects on bone structure and architecture. Contemp Clin Trials 67:56–67. doi:10.1016/j.cct.2018.02.003 29408561 PMC5877816

[31] Manson JE, Cook NR, Lee I-M, Christen W, Bassuk SS, Mora S, Gibson H, Gordon D, Copeland T, D’Agostino D, Friedenberg G, Ridge C, Bubes V, Giovannucci EL, Willett WC, Buring JE, VITAL Research Group. 2019. Vitamin D supplements and prevention of cancer and cardiovascular disease. N Engl J Med 380:33–44. doi:10.1056/NEJMoa1809944 30415629 PMC6425757

[32] LeBoff MS, Chou SH, Murata EM, Donlon CM, Cook NR, Mora S, Lee I-M, Kotler G, Bubes V, Buring JE, Manson JE. 2020. Effects of supplemental vitamin D on bone health outcomes in women and men in the VITamin D and OmegA-3 TriaL (VITAL). J Bone Miner Res 35:883–893. doi:10.1002/jbmr.3958 31923341 PMC7217747

[33] LeBoff MS, Murata EM, Cook NR, Cawthon P, Chou SH, Kotler G, Bubes V, Buring JE, Manson JE. 2020. VITamin D and OmegA-3 TriaL (VITAL): effects of vitamin D supplements on risk of falls in the US population. J Clin Endocrinol Metab 105:2929–2938. doi:10.1210/clinem/dgaa311 32492153 PMC7365686

[34] Carlberg C, Seuter S, de Mello VDF, Schwab U, Voutilainen S, Pulkki K, Nurmi T, Virtanen J, Tuomainen T-P, Uusitupa M. 2013. Primary vitamin D target genes allow a categorization of possible benefits of vitamin D₃ supplementation. PLoS One 8:e71042. doi:10.1371/journal.pone.0071042 23923049 PMC3726591

[35] Ryynänen J, Neme A, Tuomainen T, Virtanen JK, Voutilainen S, Nurmi T, de Mello VDF, Uusitupa M, Carlberg C. 2014. Changes in vitamin D target gene expression in adipose tissue monitor the vitamin D response of human individuals. Molecular Nutrition Food Res 58:2036–2045. doi:10.1002/mnfr.201400291 24975273

[36] Vukić M, Neme A, Seuter S, Saksa N, de Mello VDF, Nurmi T, Uusitupa M, Tuomainen T-P, Virtanen JK, Carlberg C. 2015. Relevance of vitamin D receptor target genes for monitoring the vitamin D responsiveness of primary human cells. PLoS One 10:e0124339. doi:10.1371/journal.pone.0124339 25875760 PMC4395145

[37] Saksa N, Neme A, Ryynänen J, Uusitupa M, de Mello VDF, Voutilainen S, Nurmi T, Virtanen JK, Tuomainen T-P, Carlberg C. 2015. Dissecting high from low responders in a vitamin D_3_ intervention study. J Steroid Biochem Mol Biol 148:275–282. doi:10.1016/j.jsbmb.2014.11.012 25448738

[38] Orkaby AR, Djousse L, Manson JE. 2019. Vitamin D supplements and prevention of cardiovascular disease. Curr Opin Cardiol 34:700–705. doi:10.1097/HCO.0000000000000675 31425172 PMC7112175

[39] Holick MF, Binkley NC, Bischoff-Ferrari HA, Gordon CM, Hanley DA, Heaney RP, Murad MH, Weaver CM. 2011. Evaluation, treatment, and prevention of vitamin D deficiency: an Endocrine Society clinical practice guideline. J Clin Endocrinol Metab 96:1911–1930. doi:10.1210/jc.2011-0385 21646368

[40] Płudowski P, Kos-Kudła B, Walczak M, Fal A, Zozulińska-Ziółkiewicz D, Sieroszewski P, Peregud-Pogorzelski J, Lauterbach R, Targowski T, Lewiński A, et al.. 2023. Guidelines for preventing and treating vitamin D deficiency: a 2023 update in Poland. Nutrients 15:695. doi:10.3390/nu15030695 36771403 PMC9920487

[41] Li Q, Chan H, Liu W-X, Liu C-A, Zhou Y, Huang D, Wang X, Li X, Xie C, Liu WY-Z, et al.. 2023. Carnobacterium maltaromaticum boosts intestinal vitamin D production to suppress colorectal cancer in female mice. Cancer Cell 41:1450–1465. doi:10.1016/j.ccell.2023.06.011 37478851

[42] Thomas RL, Jiang L, Adams JS, Xu ZZ, Shen J, Janssen S, Ackermann G, Vanderschueren D, Pauwels S, Knight R, Orwoll ES, Kado DM. 2020. Vitamin D metabolites and the gut microbiome in older men. Nat Commun 11:5997. doi:10.1038/s41467-020-19793-8 33244003 PMC7693238

[43] Adams JS, Hewison M. 2010. Update in vitamin D. J Clin Endocrinol Metab 95:471–478. doi:10.1210/jc.2009-1773 20133466 PMC2840860

[44] Haussler MR, Jurutka PW, Mizwicki M, Norman AW. 2011. Vitamin D receptor (VDR)-mediated actions of 1α,25(OH)₂vitamin D₃: genomic and non-genomic mechanisms. Best Pract Res Clin Endocrinol Metab 25:543–559. doi:10.1016/j.beem.2011.05.010 21872797

[45] Sosa-Díaz E, Hernández-Cruz EY, Pedraza-Chaverri J. 2022. The role of vitamin D on redox regulation and cellular senescence. Free Rad Biol Med 193:253–273. doi:10.1016/j.freeradbiomed.2022.10.003 36270517

[46] Meyer MB, Watanuki M, Kim S, Shevde NK, Pike JW. 2006. The human transient receptor potential vanilloid type 6 distal promoter contains multiple vitamin D receptor binding sites that mediate activation by 1,25-dihydroxyvitamin D_3_ in intestinal cells. Mol Endocrinol 20:1447–1461. doi:10.1210/me.2006-0031 16574738

[47] van de Peppel J, van Leeuwen J. 2014. Vitamin D and gene networks in human osteoblasts. Front Physiol 5:137. doi:10.3389/fphys.2014.00137 24782782 PMC3988399

[48] Terpening CM, Haussler CA, Jurutka PW, Galligan MA, Komm BS, Haussler MR. 1991. The vitamin D-responsive element in the rat bone Gla protein gene is an imperfect direct repeat that cooperates with other cis-elements in 1,25-dihydroxyvitamin D_3_- mediated transcriptional activation. Mol Endocrinol 5:373–385. doi:10.1210/mend-5-3-373 1653893

[49] Holliday LS, Patel SS, Rody WJ. 2021. RANKL and RANK in extracellular vesicles: surprising new players in bone remodeling. Extracell Vesicles Circ Nucl Acids 2:18–28. doi:10.20517/evcna.2020.02 33982033 PMC8112638

[50] Zeitelhofer M, Adzemovic MZ, Gomez-Cabrero D, Bergman P, Hochmeister S, N’diaye M, Paulson A, Ruhrmann S, Almgren M, Tegnér JN, Ekström TJ, Guerreiro-Cacais AO, Jagodic M. 2017. Functional genomics analysis of vitamin D effects on CD4^+^ T cells in vivo in experimental autoimmune encephalomyelitis. Proc Natl Acad Sci U S A 114:E1678–E1687. doi:10.1073/pnas.1615783114 28196884 PMC5338504

[51] Flórez AB, Vázquez L, Rodríguez J, Redruello B, Mayo B. 2019. Transcriptional regulation of the equol biosynthesis gene cluster in adlercreutzia equolifaciens DSM19450T. Nutrients 11:993. doi:10.3390/nu11050993 31052328 PMC6566806

[52] Zhang Q, Xu W, Xu X, Lu W, Zhao J, Zhang H, Chen W. 2021. Effects of Limosilactobacillus fermentum CCFM1139 on experimental periodontitis in rats. Food Funct 12:4670–4678. doi:10.1039/d1fo00409c 33928953

[53] Li M, Han X, Sun L, Liu X, Zhang W, Hao J. 2024. Indole-3-acetic acid alleviates DSS-induced colitis by promoting the production of R-equol from Bifidobacterium pseudolongum. Gut Microbes 16:2329147. doi:10.1080/19490976.2024.2329147 38528729 PMC10968315

[54] Cao YG, Bae S, Villarreal J, Moy M, Chun E, Michaud M, Lang JK, Glickman JN, Lobel L, Garrett WS. 2022. Faecalibaculum rodentium remodels retinoic acid signaling to govern eosinophil-dependent intestinal epithelial homeostasis. Cell Host Microbe 30:1295–1310. doi:10.1016/j.chom.2022.07.015 35985335 PMC9481734

[55] Liu PT, Stenger S, Li H, Wenzel L, Tan BH, Krutzik SR, Ochoa MT, Schauber J, Wu K, Meinken C, Kamen DL, Wagner M, Bals R, Steinmeyer A, Zügel U, Gallo RL, Eisenberg D, Hewison M, Hollis BW, Adams JS, Bloom BR, Modlin RL. 2006. Toll-like receptor triggering of a vitamin D-mediated human antimicrobial response. Science 311:1770–1773. doi:10.1126/science.1123933 16497887

[56] Misra M, Pacaud D, Petryk A, Collett-Solberg PF, Kappy M, Drug and Therapeutics Committee of the Lawson Wilkins Pediatric Endocrine Society. 2008. Vitamin D deficiency in children and its management: review of current knowledge and recommendations. Pediatrics 122:398–417. doi:10.1542/peds.2007-1894 18676559

[57] Lu Y, Chen H, Chen Y, Zhao L, Hou S. 2024. Accumulated LPS induced by colitis altered the activities of vitamin D-metabolizing hydroxylases and decreased the generation of 25-hydroxyvitamin D. Chem Biol Interact 395:110997. doi:10.1016/j.cbi.2024.110997 38588969

